# The differential effects of maternal age, race/ethnicity and insurance on neonatal intensive care unit admission rates

**DOI:** 10.1186/1471-2393-12-97

**Published:** 2012-09-17

**Authors:** Beatriz E de Jongh, Robert Locke, David A Paul, Matthew Hoffman

**Affiliations:** 1 Department of Neonatal-Perinatal Medicine, St. Christopher’s Hospital for Children, Philadelphia, PA, USA; 2 Department of Neonatology, Christiana Care Health System, Newark, DE, USA; 3 Department of Pediatrics, Thomas Jefferson University, Philadelphia, PA, USA; 4 Department of Obstetrics and Gynecology, Christiana Care Health System, Newark, DE, USA; 5 Division of Neonatal-Perinatal Medicine, St. Christopher’s Hospital for Children, Philadelphia, PA, USA

## Abstract

**Background:**

Maternal race/ethnicity, age, and socioeconomic status (SES) are important factors determining birth outcome. Previous studies have demonstrated that, teenagers, and mothers with advanced maternal age (AMA), and Black/Non-Hispanic race/ethnicity can independently increase the risk for a poor pregnancy outcome. Similarly, public insurance has been associated with suboptimal health outcomes. The interaction and impact on the risk of a pregnancy resulting in a NICU admission has not been studied. Our aim was, to analyze the simultaneous interactions of teen/advanced maternal age (AMA), race/ethnicity and socioeconomic status on the odds of NICU admission.

**Methods:**

The Consortium of Safe Labor Database (subset of n = 167,160 live births) was used to determine NICU admission and maternal factors: age, race/ethnicity, insurance, previous c-section, and gestational age.

**Results:**

AMA mothers were more likely than teenaged mothers to have a pregnancy result in a NICU admission. Black/Non-Hispanic mothers with private insurance had increased odds for NICU admission. This is in contrast to the lower odds of NICU admission seen with Hispanic and White/Non-Hispanic pregnancies with private insurance.

**Conclusions:**

Private insurance is protective against a pregnancy resulting in a NICU admission for Hispanic and White/Non-Hispanic mothers, but not for Black/Non-Hispanic mothers. The health disparity seen between Black and White/Non-Hispanics for the risk of NICU admission is most evident among pregnancies covered by private insurance. These study findings demonstrate that adverse pregnancy outcomes are mitigated differently across race, maternal age, and insurance status.

## Background

Maternal race/ethnicity, age, and socioeconomic status (SES) are important factors determining birth outcome [1-3]. Previous studies have demonstrated that, teenagers, and mothers with advanced maternal age (AMA), and Black/Non-Hispanic race/ethnicity can independently increase the risk for a poor pregnancy outcome. Similarly, public insurance has been associated with suboptimal health outcomes.

Widening neonatal mortality racial disparities with advancing maternal age is consistent with a theoretical view of aging as a “weathering” process. This hypothesis captures the ways in which social inequality may affect the health of population groups differentially and the ways in which these differences may be compounded by age; reflective of the life circumstances that promote or undermine women’s health on a population level in ways that can affect reproduction [4].

Similarly, a stress model has been proposed to explain the elevated risk of an adverse pregnancy outcome. “Stress age” is synonymous with the concepts of weathering and altered allostatic loads. The stress-age model proposes a linkage between altered birth outcomes through combined effect of chronic medical conditions and persistent exposure to stress, including racism-associated stress [5]. Despite the fact that many well-educated black women obtain prenatal care beginning in the first trimester, the relative risk of death among their offspring has increased over time [6].

This study analyzes the comparative risk of a pregnancy resulting in a newborn being admitted to the neonatal intensive care unit based upon the interaction of maternal age, race/ethnicity, and insurance status. We hypothesized that the risk of NICU admission for teenage mothers and mothers of advanced maternal age are inconsistently experienced by different maternal race/ethnicities regardless of socioeconomic status.

## Methods

The Consortium of Safe Labor Database (CSLD: n = 233844 mothers; 19 US hospitals 2002–2008) was used to determine NICU admission and maternal factors: age, race/ethnicity (defined by maternal self-report), private vs. public insurance, previous history of cesarean section, and infant birth gestational age. Maternal age was divided into three age groups; teenage defined as a maternal age between 13 and 18 years old, followed by an intermediate age group between 19 and 34 years old and an advanced maternal age group defined as 35 to 49 years of age. Similar age group distributions have been used by other authors [7,8]. Analysis was performed between three self-reported maternal race/ethnicity descriptions (White/Non-Hispanic, Black/Non-Hispanic and Hispanic) and controlled for percentage of births by maternal age, maternal insurance status, and history of previous cesarean section (c-section). To reflect the overall U.S. obstetric population, the Consortium of Safe Labor assigned a weight to each mother based on the ACOG district, maternal race/ethnicity, parity and plurality.

The Internal Review Board of Christiana Care Health System approved this study. Permission was granted by the Consortium of Safe Labor to use the information in the database.

For mothers who had several pregnancies during the CSLD data acquisition time period, a mother was included only once in the analysis. For multiple gestation pregnancies, only the first infant was utilized for NICU admission. Women with race/ethnicity different than the above criteria or with incomplete information were excluded from the analysis. Data were analyzed utilizing generalized linear models. There were no meaningful result differences whether logistic regression or generalized linear models were utilized.

## Results

**Figure 1:**
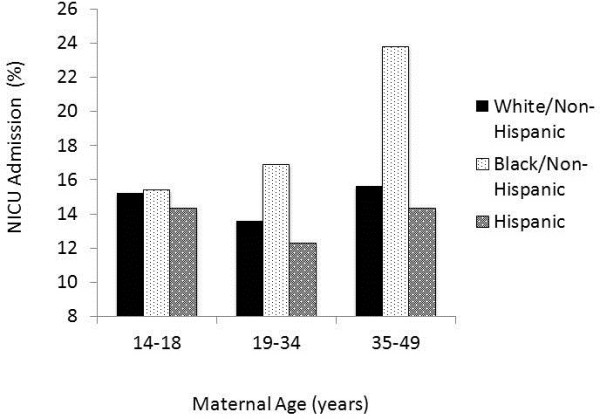
Percentage of births of each maternal age and race/ethnicity group admitted to the NICU.

The study sample included 167,160 live births with demographic information by age groups presented in Table 1. When analyzing the study population by age, advanced maternal age mothers were more likely to have a pregnancy result in a NICU admission (Figure 1). White/Non-Hispanic and Hispanic mothers had similar patterns of NICU admission rates with high rates of NICU admission with teenage pregnancies and advanced maternal age. By contrast, Black/Non-Hispanic mothers had significantly higher rates with advanced age compared to teenage and intermediately aged mothers.

**Table 1:** Patient population demographics stratified by age

**Age (years)**	**Within age group (%)**					
	**Race/Ethnicity**			**NICU admission**	**Insurance**	**Previous C-section**
	**White/Non-Hispanic**	**Black/Non-Hispanic**	**Hispanic**	**Yes**	**Private**	**Yes**
14 -18	39.7	28.5	31.8	15.0	28.7	2.1
19 – 34	64.4	14.7	20.9	13.8	59.0	14.7
35 – 49	70.8	11.5	17.8	16.3	78.8	26.2

**Figure 2:**
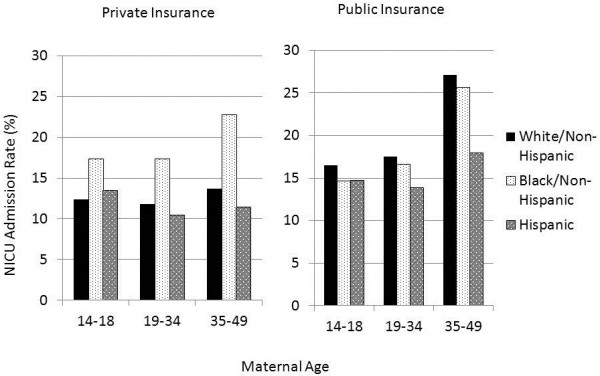
Percentage of births of each maternal age and race/ethnicity admitted to the NICU stratified by insurance status.

When stratifying for maternal age, race/ethnicity, and insurance status, there was a divergent pattern to the odds of an infant being admitted to the NICU that was dependent upon an interaction of race/ethnicity, age, and insurance status (Figure 2). Private insurance was associated with decreased odds of NICU admission between Hispanic and White/Non-Hispanic infants. In contrast, Black/Non-Hispanic mothers with private insurance had increased odds of a pregnancy resulting in a NICU admission (Table 2). Public insurance was associated with increased odds of NICU admission of the White/Non-Hispanic and Hispanic population for all three age groups. The Black/Non-Hispanic population with public insurance had lower odds of NICU admission in the teenage and intermediate age group populations than those on private insurance (Table 3). The disparity between Black/Non-Hispanic compared to the White/Non-Hispanic or Hispanic groups was greatest in the private insurance group.

**Table 2:** NICU admission by maternal age and race, controlling for maternal insurance status and history of a previous cesarean section

**Maternal race**	**Maternal age, insurance status and history of previous c-section**	**Odds ratio**	**95% Confidence interval**
White/Non- Hispanic	14 - 18 years old	.964	(0.957 - 0.971)
	19 - 34 years old	1	
	35–49 years old	1.256	(1.249 - 1.264)
	Private insurance	1	
	Public insurance	1.788	(1.778 - 1.798)
	No history of previous c-section	1	
	History of previous c-section	1.426	(1.417 - 1.435)
Black/Non-Hispanic	14 - 18 years old	.935	(0.926 - 0.945)
	19 - 34 years old	1	
	35 - 49 years old	1.476	(1.460 - 1.493)
	Private insurance	1	
	Public insurance	.944	(0.936 - 0.952)
	No History of previous c-section	1	
	History of previous c-section	1.234	(1.219 - 1.248)
Hispanic	14 - 18 years old	1.175	(1.163 - 1.186)
	19 - 34 years old	1	
	35 - 49 years old	1.204	(1.191 - 1.217)
	Private insurance	1	
	Public insurance	1.354	(1.342 - 1.365)
	No History of previous c-section	1	
	History of previous c-section	1.220	(1.206 - 1.234)

**Table 3:** OR of NICU admission by insurance, stratified by age and race/ethnicity

**Age**	**Race/ethnicity**	**Insurance OR (± 95% CI) for NICU admission**	
		**Private**	**Public**
14-18	White/Non-Hispanic	1	1.37 (1.31 – 1.41)
	Black/Non-Hispanic	1	0.79 (0.75 – 0.83)
	Hispanic	1	1.12 (1.06 – 1.18)
19-34	White/Non-Hispanic	1	1.60 (1.58 – 1.61)
	Black/Non-Hispanic	1	0.93 (0.913 – 0.947)
	Hispanic	1	1.33 (1.30 – 1.35)
35-49	White/Non-Hispanic	1	2.37 (2.32 – 2.43)
	Black/Non-Hispanic	1	1.13 (1.09 – 1.18)
	Hispanic	1	1.60 (1.54 – 1.66)

Controlled for history of previous c - section.

In each age group, Black/Non-Hispanic pregnancies with public insurance were less likely to result in a NICU admission than White/Non-Hispanic pregnancies (Table 4). Among women with public insurance, advanced maternal age was associated with higher odds of NICU admission between White/Non-Hispanic and Black/Non-Hispanics compared to the middle age and teenage maternal population. Among women with private insurance Hispanic and White/Non-Hispanics of the teenage population had increased odds of NICU admission compared to the AMA population (Table 5).

**Table 4:** OR of NICU admission by race/ethnicity, stratified by age and iInsurance

**Age**	**Race/ethnicity**	**Insurance OR (± 95% CI) for NICU admission**	
		**Private**	**Public**
14-18	White/Non-Hispanic	1	1
	Black/Non-Hispanic	1.46	0.85
		(1.37 – 1.55)	(0.82 – 0.89)
	Hispanic	1.09	0.89
		(1.03 – 1.16)	(0.86 – 0.92)
19-34	White/Non-Hispanic	1	1
	Black/Non-Hispanic	1.48	0.86
		(1.46-1.51)	(0.85-0.88)
	Hispanic	0.85	0.70
		(0.84 – 0.86)	(0.69 – 0.71)
35-49	White/Non-Hispanic	1	1
	Black/Non-Hispanic	1.74	0.86
		(1.69 – 1.78)	(0.83 – 0.90)
	Hispanic	0.79	0.55
		(0.77 – 0.82)	(0.53 – 0.57)

Controlled for history of previous c-section.

**Table 5:** OR of NICU admission by age, stratified by insurance and race/ethnicity

**Race/ethnicity**	**Maternal age**	**Insurance OR (± 95% CI) for NICU admission**	
		**Private**	**Public**
White/Non-Hispanic	14 – 18	1.17 (1.12 – 1.22)	0.99 (0.97 – 1.02)
	19 - 34	1	1
	35 - 49	1.04 (1.02 – 1.05)	1.54 (1.51 – 1.58)
Black/Non-Hispanic	14 – 18	1.15 (1.10 – 1.20)	0.97 (0.93 – 0.99)
	19 - 34	1	1
	35 - 49	1.20 (1.17 – 1.24)	1.47 (1.41 – 1.52)
Hispanic	14 – 18	1.50 (1.43 – 1.58)	1.24 (1.20 – 1.27)
	19 - 34	1	1
	35 - 49	0.97 (0.94 – 1.01)	1.18 (1.14 – 1.21)

Controlled for history of previous c-section.

In order to investigate if the observed differential in NICU admission was based on low gestation or physiologic compromise of older infants, we analyzed the data limiting gestation to infants ≥ 35 weeks. Infants < 35 weeks gestation are routinely admitted to the NICU. Infants with gestational ages ≥ 35 weeks are usually admitted for evidence of clinical compromise. When limiting the data analysis by gestational age to infants with a gestational age ≥ 35 weeks that, if healthy, would potentially be admitted to the regular newborn nursery the results remain the same.

## Discussion

The main finding of our investigation is that private insurance did not benefit all race/ethnicity groups equally. Specifically, having private insurance did not protect Black/Non-Hispanic mothers. Black/Non-Hispanic mothers with private insurance had higher NICU admission odds among teenagers and intermediate group women than age-matched women with public insurance. From our data we can not determine whether our findings directly resulted from differences in health care provision based on insurance or whether insurance was a proxy for other important factors including absence of poverty. We speculate that the etiology of the paradoxical relationship of higher NICU admission odds among Black/Non-Hispanics with private insurance compared to public insurance is likely secondary to ecological experiences, which adversely affect the mother and are potentially exacerbated by higher socioeconomic status among certain minority groups. Our study is unique in investigating NICU admission, a variable indicative of physiologic instability of the newborn and a marker for long term health care utilization, in a large multicenter study sample.

Our data suggest that among Black/Non-Hispanic mothers, private health insurance, acting as a direct effect or proxy, does not mitigate the adverse effects of life-course stressors. This finding is consistent with other studies demonstrating a lack of protective effect from improved neighborhood characteristics and income on Black/Non-Hispanic birth outcomes.

These findings are consistent with other literature that shows a wider racial gap in poor birth outcomes among women at seemingly lower risk. A stark racial disparity in the unadjusted rates of preterm birth and very low birth weight exists among women with a lifelong residence in high-income urban neighborhoods [9]. It has also been reported that the positive effects of a better socioeconomic context may be mitigated among minority women by adverse effects of racism or racial stigma [8]. In addition, Black/Non-Hispanic infants in hyper segregated areas are more likely to be preterm than in non-hyper segregated areas [10]. Higher isolation has also been associated with lower birth weight, higher rates of prematurity and higher rates of fetal growth restriction, in contrast with higher clustering being associated with more optimal outcomes [7]. There are larger racial disparities among the non-poor than the poor in the black population and among women than men [11].

Controlling the analysis for gestational age or limiting the study population to gestational age ≥ 35 weeks did not alter our findings. The lack of influence of gestational age on the findings suggests that the increased odds of NICU admission in this population are related to factors independent of premature birth. We speculate that Black/Non-Hispanic mothers with advanced age may have an increase in physiologic compromise that goes beyond the known increase in premature birth. These data are important in showing an increase in NICU admission possibly secondary to concomitant physiologic instability in infants born to Black/Non-Hispanic women. The study data provide further support of the “weathering hypothesis,” adverse maternal health may be secondary to persistent life course stressors that are not modifiable, and in fact may be exacerbated, with private insurance at the time of childbirth.

Hispanic women, when compared to the White/Non-Hispanic population, had decreased odds of NICU admission in the intermediate and advanced maternal age population for both private and public insurance. Hispanic women have lower odds for preterm birth compared to White/Non-Hispanic women. When compared to African-American women, Hispanic women are less likely than African-American women to experience any adverse pregnancy event [12]. Latina mothers in the United States have been shown to have favorable birth outcomes despite their social disadvantages. Proposed explanations for this can be classified as migratory selection processes, cultural protective factors, and increased social support [13]. There was an increased risk of NICU admission with AMA among Hispanic and White/Non-Hispanic in the public insurance group that was not seen in the private insurance group. This suggests that the “weathering hypothesis,” cumulative life course stressors affecting later health, may apply to White/Non-Hispanic and Hispanic mothers.

The results of this study were obtained without controlling for pregnancies conceived by assisted reproductive technology. Of approximately 62 million women of reproductive age in 2002, about 1.2 million, or 2%, had had and infertility related medical appointment within the previous year [14]. The risk of preterm birth is higher among infants conceived through assisted reproductive technology than for infants in the general population. This increase in risk is due, in large part, to the higher percentage of multiple-fetus pregnancies resulting from assisted reproductive technology cycles [14]. The influence of assisted reproductive technology would not be able to fully explain the differences seen due to small percentage of pregnancies conceived with this technology.

Our study has a number of important limitations. The CSLD may not be generalizable to other populations. This possibility was minimized as the database represents 19 hospitals in the United States from wide geographic regions and weighted to reflect national US nativity. Maternal insurance status may not correlate accurately with the actual socioeconomic level of each mother. There may have been other important factors influencing maternal health and wealth that we were unable to control for including maternal education level. The study also did not account for institutional policies and clinical biases that may have influenced NICU admission rates. We can not rule out the possibility that Black/Non-Hispanic mothers were more likely to deliver an infant in hospitals where NICU admission was more likely. In addition, we were only able to study insurance status at the time of birth. Length of private insurance coverage may be an important factor in estimating its effect on maternal health.

In our study, NICU admission was used as a primary outcome variable in order to investigate physiologic compromise and supplemental health care needs at birth. NICU admissions are not restricted to extremely premature infants or infants with congenital anomalies. Late preterm, term, and normal birthweight infants represent a significant percentage of NICU admissions and NICU-related health care costs [15]. In addition to the immediate health cost burden, NICU admissions are associated with increased risk for altered school-age behavior and achievement, accelerated development of health compromise, and reduced economic potential when becoming an adult. The daily NICU costs exceed $ 3,500 per infant, and it is not unusual for costs to top $ 1 million for a prolonged stay [16]. The annual societal economic burden associated with preterm birth in the United States was at least $26.2 billion in 2005, or $51,600 per infant born preterm [17].

## Conclusions

These study findings demonstrate that adverse pregnancy outcomes are mitigated differently across race/ethnicity, maternal age, and insurance status. Globally addressing the issue of teenage pregnancy or advanced maternal age, insurance status, race/ethnicity, without giving attention to the multi-layered interaction of these variables may miss important differences in outcomes. Our data indicate that the factors influencing NICU admission go beyond the narrowly defined scope of race/ethnicity, insurance status and maternal age. Life experiences, underlying health status, and intergenerational effects of fetal and early childhood programming, are potentially modifiable factors. Although expressed differently, regardless of race/ethnicity, age, or income status, all mother-fetal/infant dyads are susceptible to adverse pregnancy outcomes. Our study demonstrates that the complex interactions of race/ethnicity, insurance, and maternal age must be considered when planning programs to improve maternal health outcomes.

## Abbreviations

NICU: Neonatal intensive care unit; AMA: Advanced maternal age; SES: Socioeconomic status; CSLD: Consortium of safe labor database; ACOG: American congress of obstetricians and gynecologists.

## Competing interests

The authors of this paper have nothing to disclose.

No financial or non-financial interests to declare.

## Authors’ contributions

BEDJ, RL, DAP and MH contributed equally to this paper. MH worked with the Consortium of Safe Labor and carried out the collection of data at Christiana Care Hospital; he also contributed the knowledge of the maternal risk factors. DAP was involved in the interpretation of data, as well as in the critical revision of the manuscript prior to given it the final approval. RL and BEDJ contributed equally to the conception and design of the study, analysis and interpretation of the data; drafting the manuscript as well as the revisions needed for the final version ready to be published. MH, DAP, RL and BEDJ gave final approval of the version of the manuscript to be published. All authors read and approved the final manuscript.

## Pre-publication history

The pre-publication history for this paper can be accessed here:

http://www.biomedcentral.com/1471-2393/12/97/prepub
